# Blood flow restriction prevents muscle damage but not protein synthesis signaling following eccentric contractions

**DOI:** 10.14814/phy2.12449

**Published:** 2015-07-06

**Authors:** Mizuki Sudo, Soichi Ando, David C Poole, Yutaka Kano

**Affiliations:** 1 Department of Engineering Science, Bioscience and Technology Program, University of Electro-communicationsChofu, Tokyo, Japan; 2 Physical Fitness Research Institute Meiji Yasuda Life Foundation of Health and WelfareTokyo, Japan; 3 Department of Mechanical Engineering and Intelligent Systems, Control Systems Program, University of Electro-communicationsChofu, Tokyo, Japan; 4 Departments of Anatomy & Physiology and Kinesiology, Kansas State UniversityManhattan, Kansas

**Keywords:** Blood flow restriction, eccentric contraction, muscle damage, S6K1

## Abstract

There is a growing body of evidence to suggest that resistance training exercise combined with blood flow restriction (BFR) increases muscle size and strength in humans. Eccentric contraction (ECC) frequently induces severe muscle damage. However, it is not known whether and to what extent muscle damage occurs following ECC + BFR due to the difficulty of conducting definitive invasive studies. The purpose of this study was to examine muscle fiber damage following ECC + BFR at the cellular level. High-intensity ECC was purposefully selected to maximize the opportunity for muscle damage and hypertrophic signaling in our novel in vivo animal model. Male Wistar rats were assigned randomly to the following groups: ECC and ECC + BFR at varying levels of occlusion pressure (140, 160, and 200 Torr). In all conditions, electrical stimulation was applied to the dorsiflexor muscles simultaneously with electromotor-induced plantar flexion. We observed severe histochemical muscle fiber damage (area of damaged fibers/total fiber area analyzed) following ECC (26.4 ± 4.0%). Surprisingly, however, muscle damage was negligible following ECC + BFR_140_ (2.6 ± 1.2%), ECC+BFR_160_ (3.0 ± 0.5%), and ECC + BFR_200_ (0.2 ± 0.1%). Ribosomal S6 kinase 1 (S6K1) phosphorylation, a downstream target of rapamycin (mTOR)-phosphorylation kinase, increased following ECC + BFR_200_ as well as ECC. In contrast, S6K1 phosphorylation was not altered by BFR alone. The present findings suggest that ECC combined with BFR, even at high exercise intensities, may enhance muscle protein synthesis without appreciable muscle fiber damage.

## Introduction

Distinctive features of skeletal muscle cells are morphological and functional plasticity. Resistance training exercise is well known to increase muscle size and strength. However, resistance exercise frequently induces muscle damage, particularly when substantial eccentric contractions (ECC) are involved (Clarkson and Hubal 2002). Muscle damage is initially caused by mechanical disruption of the fiber and subsequent damage is linked to inflammatory processes and to changes in excitation-contraction coupling within the muscle (Proske and Morgan 2001; Clarkson and Hubal 2002).

There is a growing body of evidence to suggest that low-intensity resistance exercise combined with blood flow restriction (BFR) induces muscle hypertrophy as much as high-intensity resistance exercise without BFR (Laurentino et al. 2012; Martin-Hernandez et al. 2013; Vechin et al. 2015). Although the cellular mechanism(s) underlying the hypertrophy and strength gains induced by resistance exercise with BFR are not fully understood (Manini and Clark 2009; Pope et al. 2013), there is definitive evidence that the mammalian target of rapamycin complex 1 (mTORC1) signaling pathway is necessary (Gundermann et al. 2014). Furthermore, resistance exercise with BFR alters mRNA expression associated with proteolytic pathway (Manini et al. 2011), the myostatin-related gene mRNA expression (Laurentino et al. 2012) and proliferation of myogenic stem cells (Nielsen et al. 2012).

Resistance exercise combined with BFR has the potential to evoke unwanted side effects such as exacerbation of muscle damage. Given that ECC frequently leads to severe muscle damage (Clarkson and Hubal 2002), it is conceivable that reperfusion following high-intensity ECC with BFR (ECC + BFR) could augment muscle damage. However, in contrast to this view, a recent review presented the accumulating evidence for ECC + BFR not inducing muscle damage (Loenneke et al. 2014). Indeed, previous studies indicated that ECC + BFR did not elevate biomarkers associated with muscle damage in humans whatsoever (Takarada et al. 2000; Yasuda et al. 2012; Thiebaud et al. 2013, 2014). However, due, in part, to the difficulty of conducting more definitive and invasive studies it remains to be elucidated to what extent muscle damage occurs at the cellular level following high-intensity ECC + BFR and the mechanistic bases/signaling mechanisms that differentiate ECC + BFR from ECC.

Therefore, the purpose of this study was to examine muscle damage following high-intensity ECC combined with BFR at the cellular level. To this end we developed a novel animal model of in vivo ECC-induced muscle damage that we could study under control and BFR conditions. We tested the specific hypotheses that BFR would reduce or prevent ECC-induced muscle damage and would do so without compromising ECC-induced muscle protein synthesis signaling. This study has practical implications for implementing high-intensity resistance training exercise with BFR as an important therapeutic modality. Moreover, understanding the cellular mechanism(s) underlying the hypertrophy and strength gains and/or potential side effects induced by resistance exercise training combined with BFR will extend our basic knowledge of muscle function and adaptation. High-intensity ECC was specifically chosen to maximize the opportunity for muscle damage and also, therefore, to provide a substantial signal for muscle hypertrophy.

## Material and Methods

### Animals

Male Wistar rats, 12 week of age, were purchased from Japan SLC (Shizuoka Laboratory Animal Center, Hamamatsu, Japan). All rats were housed in a temperature-controlled room at 22 ± 2°C with a light–dark cycle of 12 h and received standard rat chow and water ad libitum. All procedures performed in this study conformed to the Guideline Principles for the Care and Use of Animals in the Field of Physiological Sciences (published by the Physiological Society of Japan) and were approved by the University of Electro-Communications Institutional Animal Care and Use Committee. All procedures for surgery, muscle stimulation, and muscle dissections were performed under pentobarbital anesthesia (70 mg/kg body wt. i.p.), and supplemental doses of anesthesia were administered as needed. All efforts were made to minimize the number of animals used and their suffering.

### Eccentric contraction and blood flow restriction

**Figure 1:**
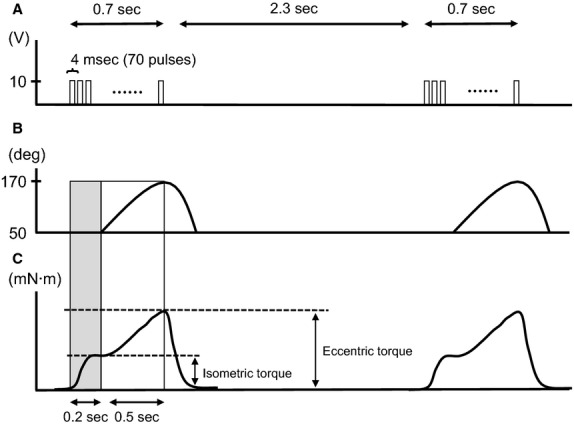
Illustration of electrical stimulation, ankle angle, and torque during eccentric contraction (ECC). (A) Electrical stimulation. Pulses of electrical stimulation continued 0.7 sec (4 msec pulse duration, 70 pulses), followed by 2.3 sec rest period. (B) Alteration in ankle angle. During the first 0.2 sec (shaded area), ankle angle was constant. For the next 0.5 sec of the stimulation, ankle joint plantar flexion was induced by the electromotor. Subsequently, the ankle joint returned to the starting position without electrical stimulation. (C) Generated torque during ECC. Vertical arrows represent isometric and eccentric torque defined in the present study.

The rats were randomly assigned to one of the following four groups: ECC with no BFR (ECC) and with BFR at 140 (ECC + BFR_140_), 160 (ECC + BFR_160_), and 200 (ECC + BFR_200_) Torr. Occlusion pressure was graded over systolic blood pressure to investigate whether occurrence of muscle damage are different depending on occlusion pressure. In all conditions, the rats were subjected to 40 repeated eccentric contractions. The right foot was attached to the clamp unit and the plate was connected to the electromotor system (Model RU-72, Motomura Systems, Tokyo, Japan). The body was placed on a hot plate with the temperature adjusted to 37°C (BWT-100, Bioresearch Center, Nagoya, Japan) in a supine position. The hair of the right lower limb was shaved for electrical stimulation. The right dorsiflexor muscles were electrically stimulated via a surface electrode (4–10 V; 100 Hz square wave stimuli; 4 msec pulse duration, 0.7 sec stimulus duration followed by a 2.3 sec rest period) under anesthesia (Fig.1A). We have previously confirmed that maximum muscle tension was achieved by this stimulation protocol (Kano et al. 2004; Sudo and Kano 2009). During the first 0.2 sec of electrical stimulation, the ankle angle remained constant (Fig.1B). We monitored ankle torque using a computer via strain-gauge-linked motor device. For the next 0.5 sec, electrical stimulation was applied simultaneously with plantar flexion by the electromotor (Fig.1B). We defined peak torque generated during this period as eccentric torque (Fig.1C). Then, during the intervening rest period, the ankle joint returned to the starting position without electrical stimulation. This cycle was repeated 40 times over 120 sec.

Blood flow was restricted by a thigh cuff (Latex cuff, overall cuff size 16 mm width, 90 mm length; D.E. Hokanson Inc, Bellevue, WA) and inflated to the designated pressure. Blood flow restriction was started 30 sec before the start of electrical stimulation and cuff pressure was released 30 sec after the last electrical stimulation.

### Histological evaluation for muscle damage

We performed histological evaluation of muscle damage from the tibialis anterior (TA) muscle. We have previously confirmed that muscle damage was prominently observed 3 days after eccentric contractions (Sudo and Kano 2009). Hence, the TA muscle was carefully dissected 3 days following ECC, ECC + BFR_140_, ECC + BFR_160_ and ECC + BFR_200_ (*N* = 5–6 for each group), and the mid-belly region was cut transversely to the long axis of the muscle. The tissue blocks were frozen rapidly in isopentane cooled by liquid nitrogen. Transverse sections of 10 *μ*m were made with a cryostat (CM1510; Leica, Wetzlar, Germany) at −20°C and stained with hematoxylin–eosin (HE) to examine the histological features of muscle damage. To avoid sampling bias, each section was subsampled at six different locations: (1) anterior-medial, (2) anterior-central, (3) anterior-lateral, (4) posterior-medial, (5) posterior-central, and (6) posterior-lateral: all regions were analyzed in the respective muscle. Muscle fiber damage was determined by a point counting method using a 30 × 40 mounted grid (i.e., 1200 points total; one square = 18 × 18 *μ*m) on microscopic fields (Kano et al. 2008; Sudo and Kano 2009). Damaged muscle fibers were defined as those with infiltration of inflammatory cells, pale staining of the cytoplasm, swollen appearance, or multiple central nuclei. The data for damaged muscle fibers were expressed as a percentage of grid squares counted (i.e., area of damaged fibers/total fiber area analyzed). The analysis of muscle fiber damage was performed by the authors and graduate students who were systematically blinded to the experimental condition for the purpose of the morphometric data collection. In additional experiments, we tested the possibility that BFR accelerates or delays the occurrence of muscle damage. Muscle damage was assessed at 1, 3, 7, and 14 days following ECC and ECC + BFR_200_ (*N* = 6 for each group). Damaged muscle fibers were defined in a similar manner as described above.

### Microvascular O_2_ partial pressure (PmvO_2_) measurements

We used a phosphorescence-quenching technique to measure PmvO_2_. This technique permits a rapid high-fidelity determination of PmvO_2_ with no invasion of the muscle vasculature (Poole et al. 2007). PmvO_2_ is thought to reflect the PO_2_ primarily within the capillary blood, and details of the PmvO_2_ measurement have been described previously (Behnke et al. 2001, 2002; Kano et al. 2011). In the present study, PmvO_2_ was monitored in the TA muscle at rest and during ECC and ECC + BFR_200_ (*N* = 4–8 for each group). At the beginning of the experiment, the oxygen phosphor R2 [palladium meso-tetra-(4-carboxyphenyl) porphine dendrimer] was infused into the carotid artery. After infusion, the rat was placed on a warming plate (37°C) in a supine position. ECC was induced in a similar manner as described above, except that electrical stimulation was applied directly to the tibial nerve via a bipolar electrode (TOG 205-053, Unique Medical, Tokyo, Japan) because skin and fascia of the TA muscle was excised and removed to permit access for the PmvO_2_ measurements.

We used a PMOD 2000 frequency domain phosphorometer (Oxygen Enterprises, Philadelphia, PA). The phosphorometer employs a sinusoidal modulation of the excitation light (524 nm), and phosphorescence (700 nm) lifetime was measured. The common end of the bifurcated light guide was placed at 2–4 mm above the center of the TA muscle. The excitation light was applied to the TA muscle at frequencies between 100 Hz and 20 kHz, which allows for phosphorescence lifetime measurements from 10 *μ*sec to ∼2.5 msec. In the single-frequency mode, 10 scans (100 msec) were used to acquire the resultant lifetime of phosphorescence and these were repeated every 2 sec. To obtain the phosphorescence lifetime, the logarithm of the intensity values was taken at each time point, and the linearized decay was fit to a straight line by least squares regression analysis. We performed curve fitting for changes in PmvO_2_ using KaleidaGraph software (version 3.6; Synergy Software, Reading, PA), as described previously (Kano et al. 2011). In the present study, we used the resting baseline value and lowest PmvO_2_ after reaching steady-state during contractions to assess the effects of ECC and ECC + BFR on PmvO_2_.

### Western blot analysis for Ribosomal S6 kinase 1

Ribosomal S6 kinase 1 (S6K1), a downstream of target of mammalian rapamycin (mTOR), phosphorylation was determined by western blot analysis (*N* = 7 for each group). The TA muscle was extracted two hours after ECC and ECC + BFR_200_. We also measured S6K1 phosphorylation after BFR at 200 Torr to assess the effects of BFR alone. The duration of BFR was the same as the ECC + BFR condition. Frozen samples were homogenized, and the homogenates were centrifuged at 8000 *g* for 15 min at 4°C, followed by the removal of the supernatant. A 10 *μ*g total protein extract was mixed with the sample buffer, and boiled, applied to SDS-polyacrylamide gel (7.5%), and then electrophoresed at 20 V for 45 min. After electrophoresis, protein was transferred to a polyvinylidene difluoride (PVDF) membrane (Nihon Millipore, Tokyo, Japan) using a semidry electrophoretic transfer system. The membrane was blocked with PBS containing 10% skimmed milk, and then incubated overnight at 4°C with the p70S6K1 Ab (#9202; Cell Signaling Technology, Beverly, MA) and phospho-p70S6K-Thr389 Ab (# 9205; Cell Signaling Technology). The next morning, blots were washed and incubated in a secondary antibody at room temperature. Bands were visualized using ECL plus Western Blotting Detection System (RPN2132, Amersham Biosciences), and chemiluminescent signals were quantified by densitometry (ATTO, Tokyo, Japan). Data are expressed as the ratio of phosphorylated to total p70S6K expression.

### Statistical analysis

All experimental data are expressed as mean ± standard error. Comparisons were performed using ANOVA with Tukey’s post hoc test. The degrees of freedom were corrected using the Huynh Feldt Epsilon when the assumption of sphericity was violated. We used nonparametric analysis to compare group differences in PmvO_2_. The level of significance was set at *P *<* *0.05.

## Results

### Isometric and Eccentric Torque

**Figure 2:**
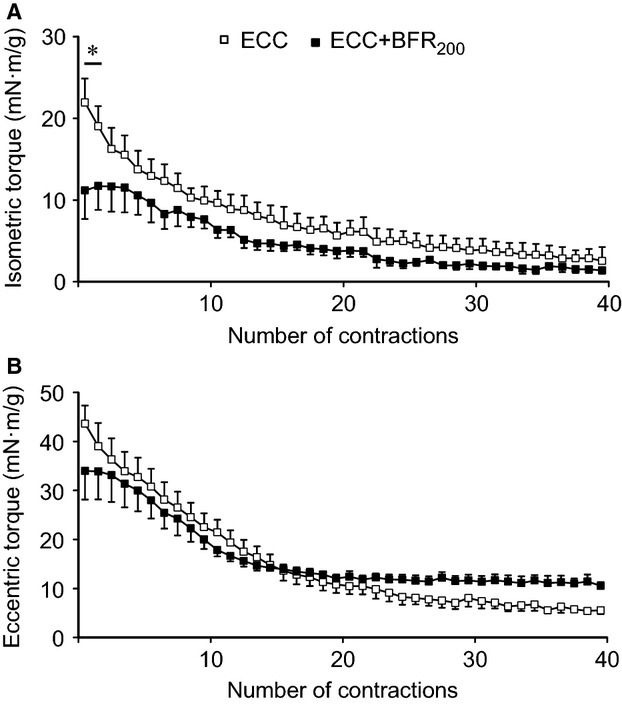
Time course of alterations in torque generated during muscle contraction by eccentric contraction (ECC) and ECC + BFR_200_. Open square shows ECC alone. Filled square shows ECC + BFR_200_. (A) isometric torque. (B) eccentric torque. *P < 0.05, versus ECC + BFR_200_.

**Figure 3:**
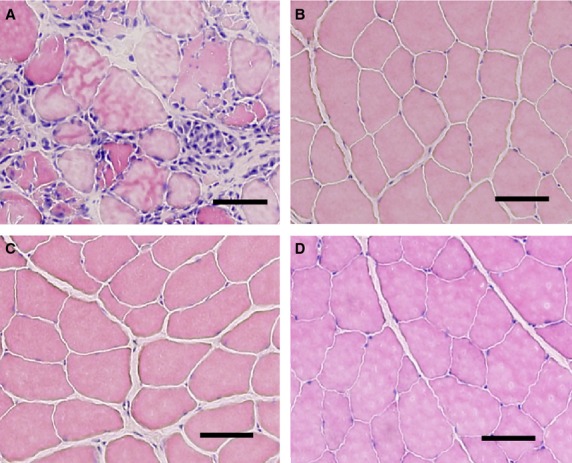
Representative photomicrographs showing muscle damage following eccentric contraction (ECC) and little-to-non following ECC + BFR. (A) ECC. (B) ECC + BFR_140_. (C) ECC + BFR_160_. (D) ECC + BFR_200_. Scale bar = 50 *μ*m.

Isometric torque was greater during ECC than ECC + BFR_200_ (*P* < 0.05) for the first two contractions (Fig.2). As the number of contractions increased isometric torque gradually decreased during both ECC and ECC + BFR_200_ (*P* < 0.001). Eccentric torque also decreased as the number of contractions increased (*P* < 0.001). However, we observed a significant interaction between eccentric torque and condition (*P* < 0.001), which is ascribed to almost constant torque during ECC + BFR_200_ during the latter part of the contractions bout. Collectively, the present results indicate that plantar flexion combined with electrical stimulation and cuff occlusion provided a sufficiently intense ECC to induce severe muscle damage (see Fig.3).

### Skeletal muscle damage following ECC and ECC + BFR

**Figure 4:**
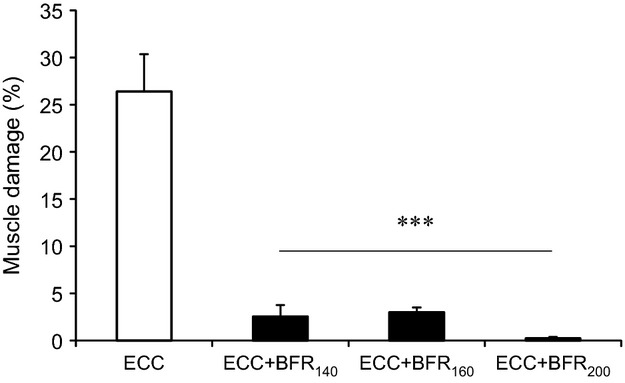
Percentage of damaged muscle following eccentric contraction (ECC) without blood flow restriction (BFR), ECC + BFR_140_, ECC + BFR_160_, ECC + BFR_200_. ***P < 0.001, versus ECC.

Focal edema and the presence of necrotic fibers invaded by mononuclear phagocytes were observed following ECC (Fig.3A). Indeed, damaged fibers were widespread in this condition. In marked contrast, we observed no signific-ant muscle damage following ECC + BFR_140_ (Fig.3B), ECC + BFR_160_ (Fig.3C), and ECC + BFR_200_ (Fig.3D). Figure4 summarizes the results of muscle damage foll-owing ECC and ECC + BFR. We observed a higher percentage of damaged fibers following ECC (26.4 ±4.0%) in comparison with the negligible damage following ECC + BFR_140_ (2.6 ± 1.2%), ECC + BFR_160_ (3.0 ±0.5%) and ECC + BFR_200_ (0.2 ± 0.1%) (*P* < 0.001 for ECC vs. all ECC + BFR).

**Figure 5:**
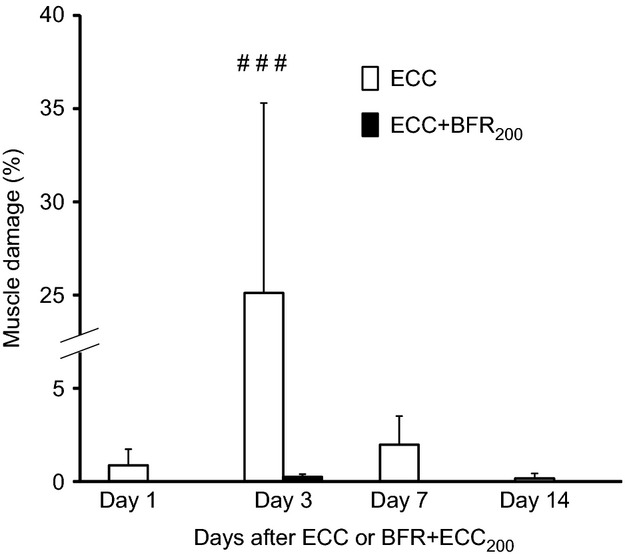
Percentage of muscle damaged at one, three, seven, and 14 days after eccentric contraction (ECC) and ECC + BFR at 200 Torr. There was practically no muscle damaged following ECC + BFR at 200 Torr. ^###^*P* < 0.001, versus Day 1, Day 7, and Day 14 after ECC alone.

As expected, the percentage of damaged fibers was higher 3 days after ECC (than at 1 or 7 days past, *P* < 0.001, Fig.5). In marked contrast practically no damaged fibers were evident following ECC + BFR_200_, irrespective of time elapsed after ECC (Fig.5).

**Figure 6:**
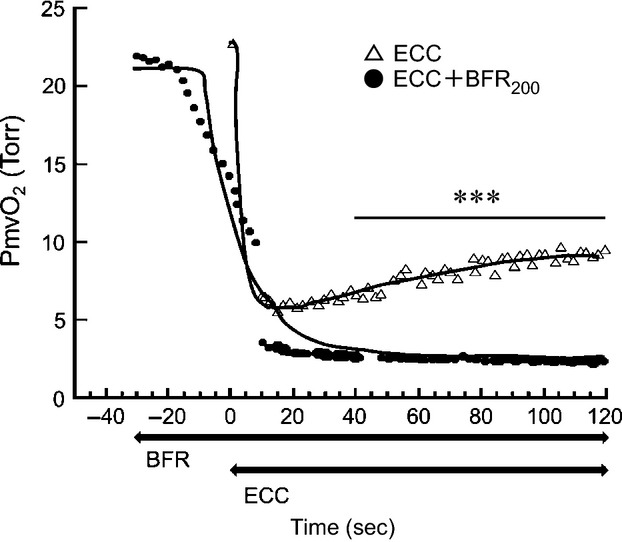
Typical example of changes in microvascular PO_2_ (PmvO_2_). Open triangle shows PmvO_2_ without blood flow restriction (BFR). Black circle shows PmvO_2_ with BFR at 200 Torr. Thick lines indicate changes in PmvO_2_ obtained by curve fitting. The horizontal axis represents time course. Contraction was initiated at time zero. Note that BFR was started 30 sec before the start of electrical stimulation. ***P < 0.001, between conditions. Note that two conditions were compared from 40 to 120 sec after the start of contractions.

### PmvO_2_ dynamics during ECC and ECC + BFR

**Figure 7:**
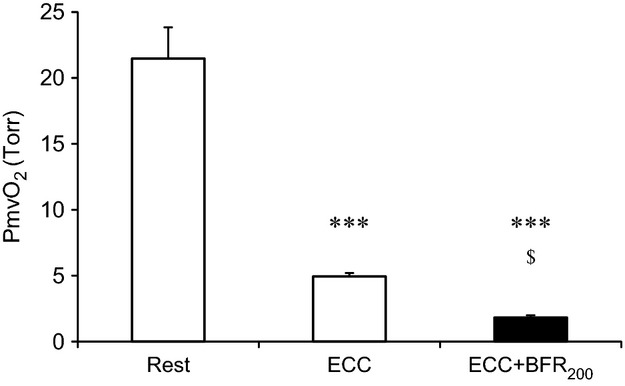
Microvascular PO_2_ (PmvO_2_) at rest, during eccentric contraction (ECC), and during ECC + BFR_200_. ***P < 0.001, versus Rest. ^$^*P* < 0.05, versus ECC.

During ECC, PmvO_2_ dropped immediately after the start of muscle contractions to reach its nadir within ∼20 sec before increasing ∼3–4 mmHg for the remainder of the bout (Fig.6). This behavior has been termed an “undershoot” and is often characteristic of fast twitch muscles. For the ECC + BFR_200_ condition PmvO_2_ began decreasing within 5–10 sec of the initiation of BFR such that, at the onset of ECC it was close to 50% lower than pre-BFR control (∼21 Torr). After the onset of ECC PmvO_2_ fell precipitously to ∼2 Torr and remained almost constant. From 40 to 120 sec of contractions PmvO_2_ was significantly lower in ECC + BFR (1.8 ± 0.2 Torr) than ECC (4.9 ± 0.3 Torr) (Fig.7).

### Western blot analysis for Ribosomal S6 kinase 1

**Figure 8:**
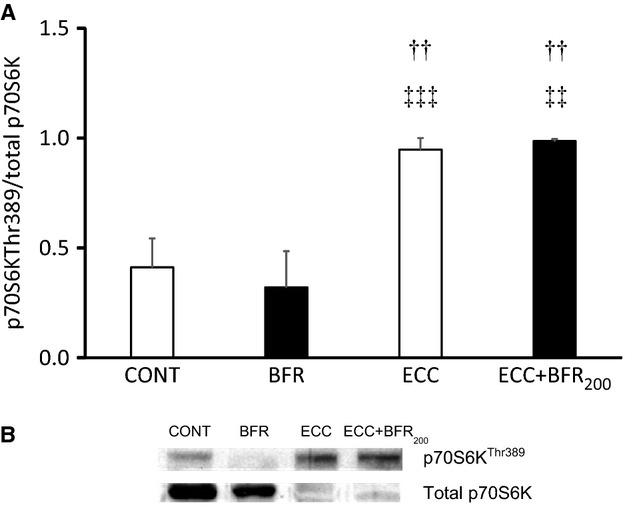
A: Phosphorylation of downstream indicator of mTOR signaling (p70S6K). (A) quantification of phosphorylated p70S6K (Thr389) to total p70S6K in control (CONT), BFR alone (BFR), ECC and ECC + BFR_200_. ^††^*P* < 0.01 versus CONT; ^‡‡^*P* < 0.01, ^‡‡‡^*P* < 0.001, versus BFR. (B) Representative western blot of phosphorylated p70S6K (Thr389) and total p70S6K contents.

Phosphorylation of p70S6K at Thr 389 was higher following ECC and ECC + BFR_200_ relative to control and BFR alone (*P* < 0.01 and *P* < 0.001) (Fig.8). BFR alone did not increase phosphorylation of p70S6K at Thr 389 under the present condition. These results indicate that ECC + BFR may produce substantial hypertrophic signaling and potential muscle growth to a similar extent as seen for ECC alone.

## Discussion

Our novel animal model permitted direct assessment of muscle fiber damage following both high-intensity ECC and ECC + BFR and provided insights into the mechanistic bases for this damage. The major original findings of this study were: (1) BFR constrained or prevented the severe muscle damage observed following ECC, and (2) S6K1 phosphorylation, indicative of the potential for muscle hypertrophy, was significantly increased following ECC + BFR_200_ as well as ECC. Additional experiments demonstrated that there was almost no muscle damage at 1, 3, 7, and 14 days after ECC + BFR_200_, which excludes the possibility that the absence of muscle damage following ECC + BFR is due to the accelerated or delayed onset of muscle damage. These findings suggest that high-intensity ECC combined with BFR may enhance muscle protein synthesis in the absence of severe muscle fiber damage potentially accounting for the enhanced hypertrophy reported after BFR exercise (Laurentino et al. 2012; Martin-Hernandez et al. 2013; Vechin et al. 2015) and suggesting that high-intensity ECC may produce substantial hypertrophic signaling and therefore potential muscle growth.

In the present study, we found that ECC + BFR generated isometric and eccentric torque, and the torque gradually decreased as the number of muscle contractions increased. These results suggest that our experimental conditions provided a sufficiently intense ECC to produce profound fatigue and also cause muscle damage. It was notable that the first few contraction cycles yielded a higher isometric torque for the ECC versus ECC + BFR condition (Fig.2). Whilst this was to be expected based on the far lower PmvO_2_ in ECC + BFR (∼50% of that in ECC) we wanted to ensure that this, in-and-of itself, did not cause the difference in muscle damage between conditions. Consequently, we mined the individual data to address this specific question. When isometric torque was compared between the animal that generated the lowest isometric torque in the ECC group and the animal that generated the highest isometric torque in the ECC + BFR group, isometric torque for the animal in the ECC group was lower than that in the ECC + BFR group. Similarly, eccentric torque of the animal that exhibited the lowest value in the ECC group was lower than that which exhibited the highest value in the ECC + BFR group. Nevertheless, muscle damage was observed in the animal that generated the lowest torque in the ECC group, whereas no apparent muscle damage was apparent in the animal that generated the highest torque in the ECC + BFR group. This dissociation between isometric torque and muscle damage provide confidence that the absence of muscle damage following ECC + BFR is not the consequence of any differences in the torque generated between ECC and ECC + BFR.

During the latter contractions, eccentric torque was almost identical in ECC and ECC + BFR. Given that ECC + BFR limits the supply of oxygen and energy substrates, enhanced energy metabolism is unlikely to account for the sustained eccentric torque. Rather, sustained eccentric torque may be associated with increased passive tension derived from a reduction in oxygen availability and energy substrates. O_2_ diffusion is expected to be decreased to a greater extent during ECC + BFR because the O_2_ gradient between capillary and cytosol must be minimal, as evidenced by the extremely low PmvO_2_. This would bring about a substantial reduction in available O_2_ and depletion of energy substrates (adenosine triphosphate [ATP] and other high-energy phosphates). ATP provides the energy for relaxation via sarco/endoplasmic reticulum Ca^2+^-ATPase (SERCA) as well as contraction at the myosin head. Thus, relaxation does not occur without ATP even though there are no more action potentials, resulting in sustained contraction which is called contracture (Barrett et al. 2012). Contracture augments muscle stiffness leading to increased passive tension (Proske and Morgan 2001). It is plausible that BFR reduced O_2_ availability compromising [ATP] resulted in contracture-increased stiffness during ECC + BFR elevating passive tension and sustained eccentric torque (Fig.2).

Several factors are potentially responsible for muscle damage following ECC (Proske and Morgan 2001; Clarkson and Hubal 2002; Butterfield 2010; Schoenfeld 2012): Among these a primary culprit is disruption of cell membrane integrity and consequent increase in [Ca^2+^]i. Elevated [Ca^2+^]i may be caused by the influx of Ca^2+^ through stretch-activated ion channels (SAC) localized on the cell membrane (Yeung and Allen 2004; Allen et al. 2005; Zhang et al. 2012). Indeed, SAC blockers abolish or substantially reduce accumulation of [Ca^2+^]i following ECC in vivo (Yeung and Allen 2004; Allen et al. 2005; Yeung et al. 2005; Sonobe et al. 2008). That muscle damage was not observed following ECC + BFR (Figs.4, 5) suggests that any substantial (i.e., damaging) increase in [Ca^2+^]i was prevented. Given that the SAC opens in response to mechanical deformation, the absence of muscle damage suggests that mechanical deformation may have been suppressed during ECC + BFR. This notion is supported by the lack of reduced eccentric torque during the latter part of the contractions bout in the ECC + BFR condition; possibly due to increased passive tension derived from contracture. Future studies should elucidate the specific physiological mechanisms underlying the suppression of ECC-induced muscle damage by BFR.

Increasing evidence suggests that low-intensity resistance training exercise combined with BFR increases muscle size and strength as much as conventional resistance exercise with high loads (Laurentino et al. 2012; Martin-Hernandez et al. 2013; Vechin et al. 2015). A recent study demonstrated that the mTORC1 signaling pathway is necessary (Gundermann et al. 2014). Indeed, S6K1 phosphorylation, a downstream mTOR-phosphorylation kinase, is increased after resistance training with BFR (Fujita et al. 2007; Fry et al. 2010). In the present study, S6K1 phosphorylation increased substantially following ECC + BFR_200_ to an extent similar to that found with ECC alone. In contrast, S6K1 phosphorylation was not increased following BFR alone. These results suggest that high-intensity ECC + BFR may produce substantial hypertrophic signaling and potential muscle growth in the absence of muscle damage, and that muscle contractions are necessary to enhance protein synthesis at least under the experimental conditions herein. The present results demonstrate that ECC + BFR activates the mTOR-S6K1 signaling cascade in the absence of severe muscle damage, which supports the notion that even high-intensity exercise combined with BFR can yield potentially beneficial muscle adaptations without any negative consequences of attendant muscle damage (Loenneke et al. 2014).

One may argue that the present results are not consistent with the previous study showing that inhibition of SAC during ECC attenuated S6K1 phosphorylation (Spangenburg and McBride 2006). However, in this previous study, S6K1 phosphorylation still increased following ECC after the SAC inhibition (Spangenburg and McBride 2006), indicating that inhibition of SAC does not block S6K1 phosphorylation completely. Herein, we found that increases in S6K1 phosphorylation were similar between ECC and ECC + BFR.

In the present investigation, muscle contractions with cuff occlusions were performed under anesthesia. Hence, motor unit recruitment patterns were undoubtedly different from those evoked during voluntary muscle contractions. However, as far as we know, this animal model is the first that is capable of examining the effects of ECC + BFR on muscle fiber damage at the cellular level. The significance of this study lies in that muscle damage was evaluated directly at the cellular level in vivo following ECC + BFR. Finally, it should be noted that the present findings do not mean that there are no potential side effects of BFR, including adverse cardiovascular responses, blood clotting, and nerve damage. Furthermore, we did not assess alterations in hypertrophy and muscle strength after long-term ECC + BFR. Longitudinal studies are necessary to assess potential side effects and to validate/better understand the experimental model presented in the present investigation.

## Conclusions

This study examined muscle damage following high-intensity ECC + BFR at the cellular level. S6K1 phosphorylation increased following ECC + BFR supporting that high-intensity ECC combined with BFR may produce substantial hypertrophic signaling and potential muscle growth without invoking the severe muscle fiber damage associated with ECC.
